# Nanoparticle-Formulated Curcumin Prevents Posttherapeutic Disease Reactivation and Reinfection with *Mycobacterium tuberculosis* following Isoniazid Therapy

**DOI:** 10.3389/fimmu.2017.00739

**Published:** 2017-06-30

**Authors:** Sultan Tousif, Dhiraj Kumar Singh, Sitabja Mukherjee, Shaheer Ahmad, Rakesh Arya, Ranjan Nanda, Anand Ranganathan, Maitree Bhattacharyya, Luc Van Kaer, Santosh K. Kar, Gobardhan Das

**Affiliations:** ^1^Special Centre for Molecular Medicine (SCMM), Jawaharlal Nehru University, New Delhi, India; ^2^Department of Biochemistry, University of Calcutta, Kolkata, India; ^3^International Centre for Genetic Engineering and Biotechnology, New Delhi, India; ^4^School of Biotechnology, KIIT University, Bhubaneswar, Odisha, India; ^5^Department of Pathology, Microbiology and Immunology, Vanderbilt University School of Medicine, Nashville, TN, United States

**Keywords:** tuberculosis, immunotherapy, disease relapse, helper T cells, AICD, hepatotoxicity

## Abstract

Curcumin, the bioactive component of turmeric also known as “Indian Yellow Gold,” exhibits therapeutic efficacy against several chronic inflammatory and infectious diseases. Even though considered as a wonder drug pertaining to a myriad of reported benefits, the translational potential of curcumin is limited by its low systemic bioavailability due to its poor intestinal absorption, rapid metabolism, and rapid systemic elimination. Therefore, the translational potential of this compound is specifically challenged by bioavailability issues, and several laboratories are making efforts to improve its bioavailability. We developed a simple one-step process to generate curcumin nanoparticles of ~200 nm in size, which yielded a fivefold enhanced bioavailability in mice over regular curcumin. Curcumin nanoparticles drastically reduced hepatotoxicity induced by antitubercular antibiotics during treatment in mice. Most interestingly, co-treatment of nanoparticle-formulated curcumin along with antitubercular antibiotics dramatically reduced the risk for disease reactivation and reinfection, which is the major shortfall of current antibiotic treatment adopted by Directly Observed Treatment Short-course. Furthermore, nanoparticle-formulated curcumin significantly reduced the time needed for antibiotic therapy to obtain sterile immunity, thereby reducing the possibility of generating drug-resistant variants of the organisms. Therefore, adjunct therapy of nano-formulated curcumin with enhanced bioavailability may be beneficial to treatment of tuberculosis and possibly other diseases.

## Introduction

Tuberculosis (TB) remains a major global health problem that claims ~2 million lives and causes 9.6 million infections every year (1). More than 90% of people exposed to *Mycobacterium tuberculosis* (*M.tb*) remain asymptomatic throughout their life, and only ~10% display active TB disease, especially when exposed to profound immune perturbation such as during infection with human immunodeficiency virus, treatment with immune-suppressive drugs, and in diseases such as diabetes that are associated with immune deficiency (2–4). In the majority of infections, the immune system is sufficient to confine the harbored organisms within organized cellular structures called granulomas and establish latent infection (2–4). The vast majority of infected individuals successfully eliminates the harbored *M.tb* organisms and remains resistant to TB throughout life (5, 6). Taken together, these observations suggest that the immune system can effectively control *M.tb* infection. Findings from animal models, as well as patient data, have clearly established that IFN-γ-producing Th1 cells play a pivotal role in host protection against TB (7–9). In contrast, IL-4-producing Th2 cells and regulatory T (Treg) cells promote disease progression by inhibiting Th1 responses (9–13). However, we recently showed that Stat-6^−/−^TGF-βRIIDN animals, which are devoid of Th2 cells and contain reduced numbers of functional Treg cells, mount exuberant inflammatory Th1 responses and display suppressed bacterial burden, yet this response was not sufficient to induce sterile immunity (9). Therefore, immunotherapy directed toward promoting Th1-mediated inflammatory responses may not be sufficient for achieving sterile immunity.

The internationally accepted therapy for TB, Directly Observed Treatment Short-course (DOTS) is effective but has multiple serious shortfalls (1, 14–17). Even though called short-course, the regimen involves 6–9 months of treatment for regular TB and 12–24 months or more for drug-resistant TB. This poses substantial risks for the generation of drug-resistant variants of the mycobacterial organisms. In addition, this mixture of antibiotics shows marked toxicity, often leading to treatment cessation by patients (18). Most importantly, DOTS-treated patients exhibit posttreatment susceptibility to reinfection and disease reactivation (19–21). It is now evident that antitubercular antibiotics such as isoniazid (INH) cause toxicity to antigen-activated T cells during treatment, which might contribute to posttreatment hypersusceptibility to disease reactivation and reinfection (22). Therefore, addition of an immunomodulator to antibiotic therapy that reduces the treatment length and restores host protective immunity is desired not only for improving the treatment outcome but also to reduce the possibility of generating multiple and extremely drug-resistant (MDR and XDR) variants of TB (23–25).

Tuberculosis is a complex disease with two components: replicating microorganisms and inflammation. Therefore, addition of anti-inflammatory drugs along with antibiotics may be beneficial. In fact, it has been shown that addition of steroids to conventional antitubercular antibiotics yields improved therapeutic efficacy (26). In addition, clofazimine, an inhibitor of the Kv1.3 potassium channel, is an anti-inflammatory compound widely used for the treatment of leprosy (27–31), and also exhibits treatment efficacy against TB in an animal model (32–34). Similarly, curcumin is also an inhibitor of Kv1.3 (35) and exhibits therapeutic benefits in several inflammatory and infectious diseases (36–38). Curcumin analogs have been reported to show promising *in vitro* antimycobacterial activity against drug-resistant strains of *M.tb* (39, 40). Recently, curcumin was shown to augment mycobacterial killing in host macrophages by inducing apoptosis (41). In addition, curcumin potently inhibits hepatotoxicity (38), which is a concurrent problem in many antibiotic therapies that include TB therapy (18). Taken together, these data have provided strong evidence that curcumin is an excellent anti-inflammatory immunomodulator and has therapeutic potential in a variety of diseases. Despite the myriad of activities reported, curcumin is yet to be approved as a therapeutic agent due to bioavailability issues. Many clinical and experimental studies have clearly established that regular curcumin has very low bioavailability and is thus unsuitable for prolonged use (42, 43). Low systemic bioavailability of curcumin after oral dosing has been consistent throughout decades of studies in preclinical models as well as human clinical trials (44–46). This low systemic bioavailability has been attributed to its poor intestinal absorption, rapid metabolism, and rapid systemic elimination (46, 47). The plasma concentrations of curcumin were found to be surprisingly low even with oral dosing of up to 100 mg/kg in mice (48) and 2 g/kg in rats and humans (49). Therefore, enhancing the bioavailability of curcumin is the main challenge for its applications in therapeutics.

To overcome this limitation of curcumin, we have generated nanoparticle-formulated curcumin adopting a simple one-step process, which has fivefold increased bioavailability than regular curcumin, and tested its efficacy in a murine TB model. We show that curcumin nanoparticles drastically reduce the hepatotoxicity induced by the antitubercular drug isoniazid (INH). Furthermore, curcumin nanoparticles enhanced T cell-mediated immunity and prevented post-therapy susceptibility to reinfection and reactivation of the disease. In addition, curcumin nanoparticles significantly reduced the length of treatment needed for attaining sterile infection, and therefore is expected to reduce the risk for the generation of MDR and XDR variants of TB. Taken together these data indicate that curcumin nanoparticles are a promising adjunct to conventional antibiotic therapy of TB, and possibly other disease therapies.

## Materials and Methods

### Mice

Six- to eight-week-old female BALB/c mice maintained at our specific pathogen-free animal facility at the International Centre for Genetic Engineering and Biotechnology (ICGEB, New Delhi, India) were used throughout the study. All animal experiments were conducted in accordance with guidelines approved by the Institutional Animals Ethics Committee (approval ID: ICGEB/AH/2011/2/IMM-30) of ICGEB, New Delhi, India and Department of Biotechnology (DBT) guidelines, Government of India. At the relevant times after infection with *M.tb*, all mice were humanely sacrificed by asphyxiation in carbon dioxide according to institutional and DBT regulations.

### Preparation of Curcumin Nanoparticles

Four grams of curcumin (Sigma, USA) were dissolved in 1 L of distilled ethanol at room temperature and filtered to obtain a clear solution. This solution was then stirred in a high-speed homogenizer (T 25 digital ULTRA-TURAX, IKA, USA) at 12,000–15,000 RPM and a required volume of Milli Q water containing 0.1% citric acid (Merck, India) was added to it slowly over a period of 30 min until the ethanol concentration became 40% (V/V) and curcumin nanoparticles started to precipitate from the solution. Then the entire suspension was homogenized over ice in a high-pressure homogenizer (Avestin C5 High Pressure Homogeniser BPS, UK) at 30,000 PSI for 20 cycles. The aqueous suspension was then made to 0.1% polysorbate 80 (Sigma, USA) and homogenized at 12,000–15,000 RPM (T 25 digital ULTRA-TURRAX, IKA, USA) again for 1 h and filtered. The filtered slurry was dried at 60°C in an oven to get nano curcumin powder. Particle size was determined by a high resolution transmission electron microscope (JEM 2100F, JEOL, USA).

### *In Vivo* Bioavailability of Curcumin Nanoparticles

For determination of bioavailability, the same group of 12 female 6- to 8-week-old BALB/c mice was used for all the experiments. After completion of each experiment and bleeding, the animals were rested for 7 days before the start of the next experiment. Pilot experiments were done to validate the dose to be given to each animal so that curcumin can be detected in the plasma. The method of extraction and the number of times it has to be extracted for near complete extraction of the analyte was standardized before the start of the experiment. The mice were given intraperitoneal injections of natural curcumin or curcumin nanoparticles suspended in 100 µL PBS at 100 mg/kg body weight in two different experiments. This dose was found to be the minimum dose of natural curcumin that has to be injected through the intraperitoneal route for detection of curcumin in the plasma. Therefore, it was used for both the natural curcumin as well as the nanoparticle-formulated curcumin experiments for determination of bioavailability. Blood was taken at different time points after intraperitoneal injection into each mouse, and samples were pooled, plasma was prepared for each time point, and curcumin was extracted using a published procedure (50). Briefly, 350 µL of plasma prepared from the blood collected at different time points from 12 female 6- to 8-week-old BALB/c mice injected by the intraperitoneal route with either natural curcumin or nanoparticle-formulated curcumin was used for extracting curcumin with 10 mL of a mixture of ethyl acetate (Spectrochem, India): isopropanol (Merck, India), 9:1 (V/V) three times. The organic layer was separated and dried in an oven at 60°C over a stream of nitrogen, and curcumin was quantified by GC-MS as described below.

The half-lives (*T*_1/2_) of the analytes under their respective experimental conditions and dosage were calculated in GraphPad Prism 6 software by the method of interpolating unknowns from a given non-linear regression based on “plateau followed by one phase decay” equation. For the GC-MS analyses of nanoparticle-formulated curcumin, the dried samples were dissolved in 100 µL of dehydrated methanol (Sigma), vortexed, centrifuged, and transferred to 2 mL Agilent vials with 200 µL glass inserts. For the absolute quantitation, the standard curves of curcumin were prepared with concentration ranges of 0.025, 0.05, 1, 2, 3, 4, 5, 6, 8, 10, and 12 mg/mL. GC-MS analysis were carried out on an Agilent 7890A GC with Restek RTX5 capillary column (crossbond—5% diphenyl/95% dimethyl polysiloxane, 30 m× 0.25 mm, 0.25 µm film thickness) interfaced to an Agilent 7000 triple quadrupole mass spectrometer. The initial oven temperature was held at 60°C for 1 min, increased up to 180°C with a heating rate of 10°C/min; then the column temperature was programmed from 180 to 300°C by a heating rate of 50°C/min and held at this temperature for 1 min with total runtime of 16.4 min with solvent delay of 6 min and post runtime of 5 min. The carrier gas was He with a flow rate of 3 mL/min. The MS source temperature and MS quad temperature were adjusted to 230 and 150°C, respectively. Sample size injected was 1.0 µL in splitless mode. GC-MS spectra were recorded at 70 eV ionization voltage, and the mass range was from *m*/*z* 33 to 450 amu. Identification of compounds and interpretation of mass spectrum (GC-MS) was conducted using the database of National Institute of Standard and Technology.

### *M.tb* Low-Dose Aerosol Infection Model

This assay was performed as per the protocol described earlier by Tousif et al. (22).

### Drug Administration

50 mg/L of isoniazid (Sigma, USA) was administered *ad libitum* in drinking water daily, and 10 mg/kg body weight of curcumin nanoparticles suspended in 100 µL of PBS was administered intraperitoneally daily.

### Colony Forming Unit Estimation

This assay was performed as per the protocol described earlier by Tousif et al. (22).

### Flow Cytometry: Surface and Intracellular Staining

Spleens and lungs were isolated from mice and macerated by frosted slides in 10% RPMI 1640 (Gibco, Invitrogen, UK) to prepare a single cell suspension. We followed the protocol for surface and intracellular staining for flow cytometry as described earlier by Tousif et al. (22).

### Antibodies and Reagents

We used the following antibodies: anti-CD4 (clone: GK1.5)-FITC, -PerCP-Cy5, or -APC, anti-CD8 (clone: 53-6.7)-FITC, -PerCP-Cy5, or -APC, anti-CD44 (clone: IM7)-APC, anti-BrdU (clone: Bu20a)-PE, 7AAD, anti-IFN-γ (clone: XMG1.2)-APC, anti-IL-17 (clone: TC11-18H10.1)-PE, anti-IL-4 (clone: 11B11)-PE, anti-IL-6 (clone: MPS-20F3)-PE, anti-IL-12 (clone: C15.6)-PE, anti-IL-22 (clone: Poly5164)-PE, anti-IL-10 (clone: JES5-16E3)-PE, anti-IL-9 (clone: MH9A4)-PE, anti-TNF-α (clone: MP6-XT22)-PE (all from Biolegend, USA), anti-Active Caspase-3 (clone: C92-605)-FITC or -PE (from BD Pharmingen™, USA), and anti-CD69 (clone: H1.2F3)-PE (from eBiosciences, USA).

### *In Vitro* and *In Vivo* T Cell Proliferation Assay

These assays were performed as per the protocol described earlier by Tousif et al. (22).

### Hepatotoxicity Assays

Serum ALT, AST, and ALP levels were used as indicators of hepatotoxicity. These assays were performed by using diagnostic kits obtained from Span Diagnostic Limited (India) in accordance with the manufacturer’s protocol.

### Activation of OT-II TCR Tg T Cells in the Presence of Ovalbumin (OVA) Peptide

Splenocytes isolated from OT-II TCR transgenic mice were cultured in the presence of 1 µg/mL OVA peptide along with 1 µg/mL INH and 10 µg/mL curcumin nanoparticles for 72 h. The cells were then stained and analyzed for T cell activation by FACS.

### Statistical Analysis

All data were analyzed by Excel 2007. In all the figures, mean values were calculated with SD unless stated otherwise. For all statistical analyses Student’s *t*-test was performed to compare two groups, and *p* < 0.05 was considered significant.

## Results and Discussion

### A Nanoparticle Formulation of Curcumin Extends Its Bioavailability *In Vivo*

**Figure 1:**
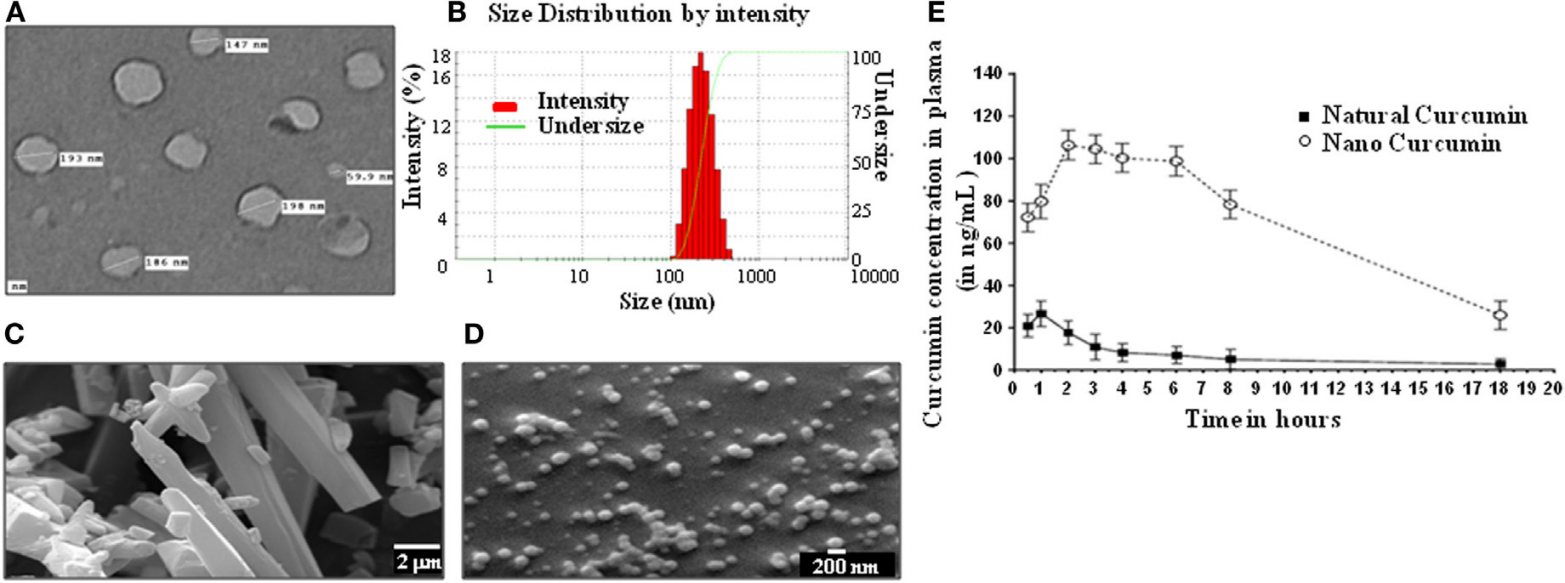
Physical properties and bioavailability of curcumin nanoparticles. **(A)** Image of curcumin nanoparticles taken with a transmission electron microscope (JOEL 2100F) showing particles having a size distribution of 59.9–198 nm (average size of 160 nm). **(B)** Dynamic light scattering of curcumin nanoparticles, showing unimodal size distribution with mean diameter of 213.3 nm and polydispersity index of 0.111. Analysis was performed (scattering angle = 90°, laser wavelength = 632.8 nm) on a 256 channel Photocor-FC (Photocor Inc., USA) operated in the multi-tau mode (logarithmically spaced channels). **(C)** Images of normal curcumin crystals having a size ranging from 1 to >20 µm, as seen by a scanning electron microscope (EM-LEO 435, Carl Zeiss SMT Inc., NY, USA). **(D)** Images of curcumin nanoparticles as seen by a scanning electron microscope (EM-LEO 435, Carl Zeiss SMT Inc., NY, USA), showing spherical morphology and average size of 200 nm. **(E)** *In vivo* bioavailability of curcumin nanoparticles. 100 mg/kg of natural curcumin or curcumin nanoparticles was suspended in PBS and injected intraperitoneally into mice. Blood was collected from these mice at different time points, plasma was prepared, and curcumin was extracted using the method described in Section “Materials and Methods,” and curcumin was quantitated by GC-MS for determining bioavailability.

Curcumin is a well-known immunomodulator that is efficacious against several indications. However, the bioavailability of regular curcumin is poor and not suitable for treatment of diseases such as TB. Hence, we have generated a nanoparticle formulation of curcumin, which has a uniform size of ~200 nm as measured by electron microscopy (Figures 1A–D), and possesses an extended bioavailability (Figure 1E). Curcumin nanoparticles, when injected by intraperitoneal route to mice at 100 mg/kg body weight, showed about five times improved bioavailability of curcumin at 2 h postinjection with peak plasma concentration (*C*_MAX_) of 106 ng/mL and a half life (*T*_1/2_) of 13.1 h in comparison to natural curcumin which peaked at 1 h with *C*_MAX_ of 26 ng/mL and *T*_1/2_ of 2.65 h (Figure 1E).

### Curcumin Nanoparticles Augment Antitubercular Treatment by Accelerating Pathogen Clearance

**Figure 2:**
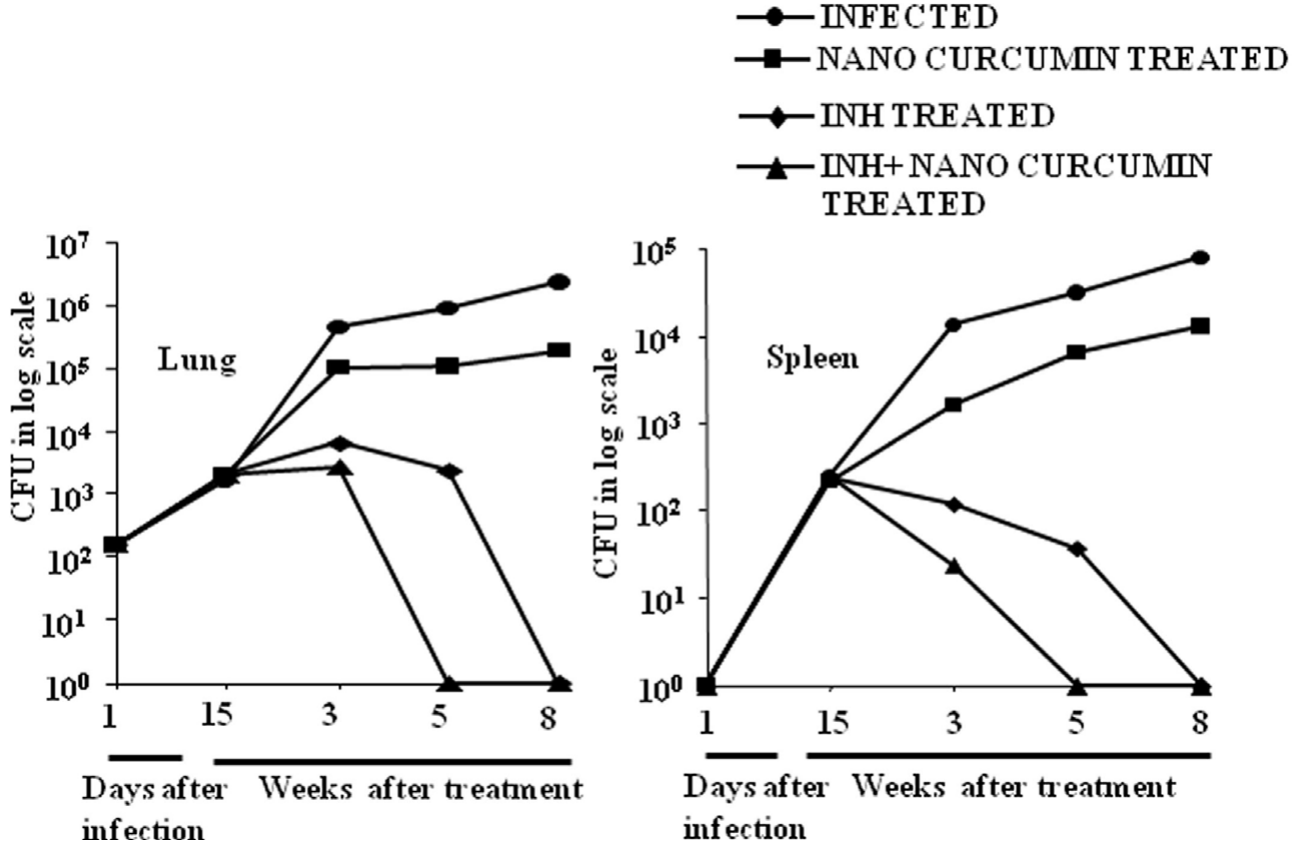
Curcumin nanoparticles augment INH therapeutic efficacy and accelerate pathogen clearance. Mice infected with a low-dose aerosol inoculum [~110 colony forming unit (CFU)/mice] of *Mycobacterium tuberculosis* H37Rv were sacrificed at various time points, and lungs were harvested, homogenized in 0.2 µm filtered PBS containing 0.05% Tween 80, and plated onto 7H11 Middle brook plates containing 10% oleic acid, albumin, dextrose, and catalase. Undiluted, 10-fold diluted, and 100-fold diluted lung and spleen cell homogenates were plated in duplicate on the above 7H11 plates and incubated at 37°C for 21 days. Colonies were counted, and CFU was estimated. Data shown here are representative of three independent experiments. Each CFU experiment has been carried out with three mice per experiment.

As curcumin is an excellent immunomodulator, anti-inflammatory agent and antioxidant, and exhibits efficacy against various diseases, we sought to test if nanoparticle-formulated curcumin has a beneficial effect on *M.tb* clearance when added as an adjunct therapy. We treated *M.tb* infected animals with INH or with INH plus nanoparticle-formulated curcumin. We observed that curcumin nanoparticles by themselves inhibited the growth of *H37Rv* by at best 1-log in a murine model of TB, whereas animals treated with both curcumin nanoparticles and INH exhibited a dramatically accelerated clearance of the microorganisms in both lung and spleen (Figure 2). Moreover, curcumin treatment also restored the INH-induced suppression in antigen-specific cytokine responses (Figure S1 in Supplementary Material). These observations indicated that nanoparticle-formulated curcumin by itself is not only antimycobacterial, but also enhances *M.tb* clearance by promoting antitubercular immunity and thereby reduces the duration of therapy.

### Curcumin Nanoparticles Prevent Disease Reactivation and Reinfection

**Figure 3:**
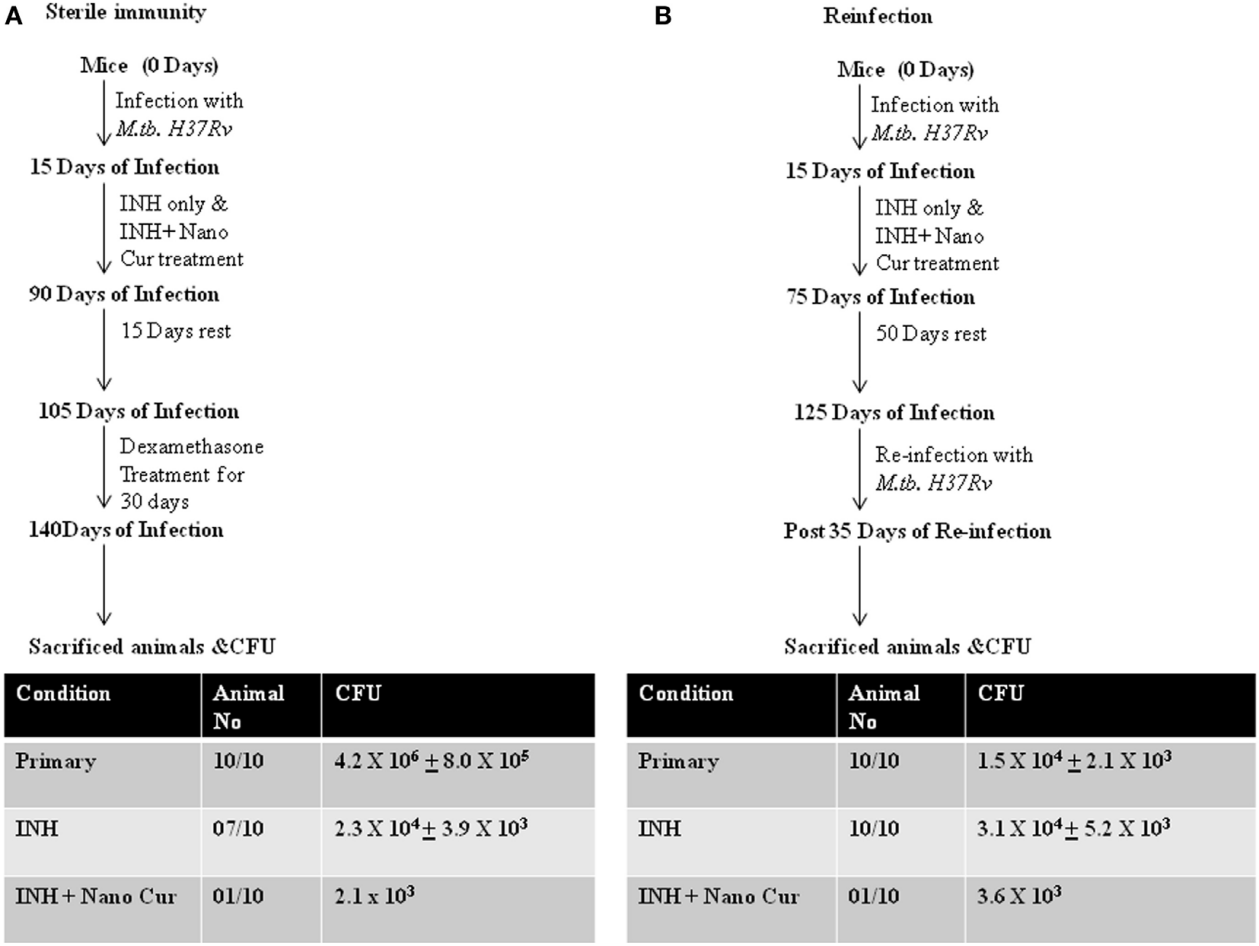
Combined therapy with INH and curcumin nanoparticles induces sterile immunity that protects against recurrent disease both *via* reactivation or reinfection. **(A)** Treatment with curcumin nanoparticles and isoniazid provides superior sterilizing activity against latent tuberculosis when compared to conventional isoniazid treatment. Mice infected with *Mycobacterium tuberculosis* strain H37Rv following the low-dose aerosol infection model [~110 colony forming unit (CFU)/mice] were treated with 50 mg/L isoniazid-administered *ad libitum* (in the drinking water) daily or 50 mg/L isoniazid-administered *ad libitum* along with 10 mg/kg body weight curcumin nanoparticles administered intraperitoneally daily until complete eradication of infection, i.e., for 75 days. These mice were then rested for 15 days followed by dexamethasone treatment (0.8 mg/kg body weight administered intraperitoneally) three times per week for 30 days. Mice were then sacrificed, and CFU was analyzed from lung homogenates to estimate the reactivation of latent mycobacteria. **(B)** Isoniazid treatment sensitizes animals to *M.tb* reinfection, which can be overcome by combined therapy with curcumin nanoparticles. Mice infected with *M.tb* strain H37Rv following the low-dose aerosol infection model (~110 CFU/mice) were treated with 50 mg/L isoniazid-administered *ad libitum* daily or 50 mg/L isoniazid-administered *ad libitum* along with 10 mg/kg body weight curcumin nanoparticles administered intraperitoneally daily until after complete eradication of infection, i.e., for 60 days. These mice were then rested for 50 days followed by reinfection with *M.tb* using the same dose and protocol as for the primary infection. These mice were then sacrificed after 35 days, and lungs were harvested to evaluate the frequency of reinfection.

From the preceding sections, it is clear that addition of curcumin to the conventional TB therapy dramatically enhances *M.tb* clearance by promoting antitubercular immunity. Therefore, to determine whether elevated antitubercular immunity promoted by curcumin nanoparticles helps in attaining sterile immunity and restores host protective memory responses we performed reactivation and reinfection experiments. We found that animals receiving adjunct treatment of curcumin nanoparticles were dramatically protected from reactivation of TB (Figure 3A). This observation suggested that addition of curcumin, an immunomodulator, is beneficial in attaining sterile immunity in the majority of subjects at a faster pace. It is highly likely that the heightened immune response attained by the addition of curcumin nanoparticles to the treatment regimen might have restored memory immune responses that protect animals from future infections. To test this possibility, we performed reinfection experiments. We observed that animals previously treated with curcumin nanoparticles are largely resistant to *M.tb* infection, indicating that a strong host protective immune response from the previous infection is restored. In sharp contrast, animals that previously received only INH displayed increased susceptibility to reinfection (Figure 3B). We further found that animals previously treated with both curcumin nanoparticles and INH exhibited drastically increased Th1 responses compared with animals that only received INH (Figure S2 in Supplementary Material). This finding is in agreement with recent studies demonstrating that Th1 responses are required and sufficient for host protective immune responses against *M.tb* infection (8).

### Curcumin Nanoparticles Protect Animals from INH-Induced Hepatotoxicity

**Figure 4:**
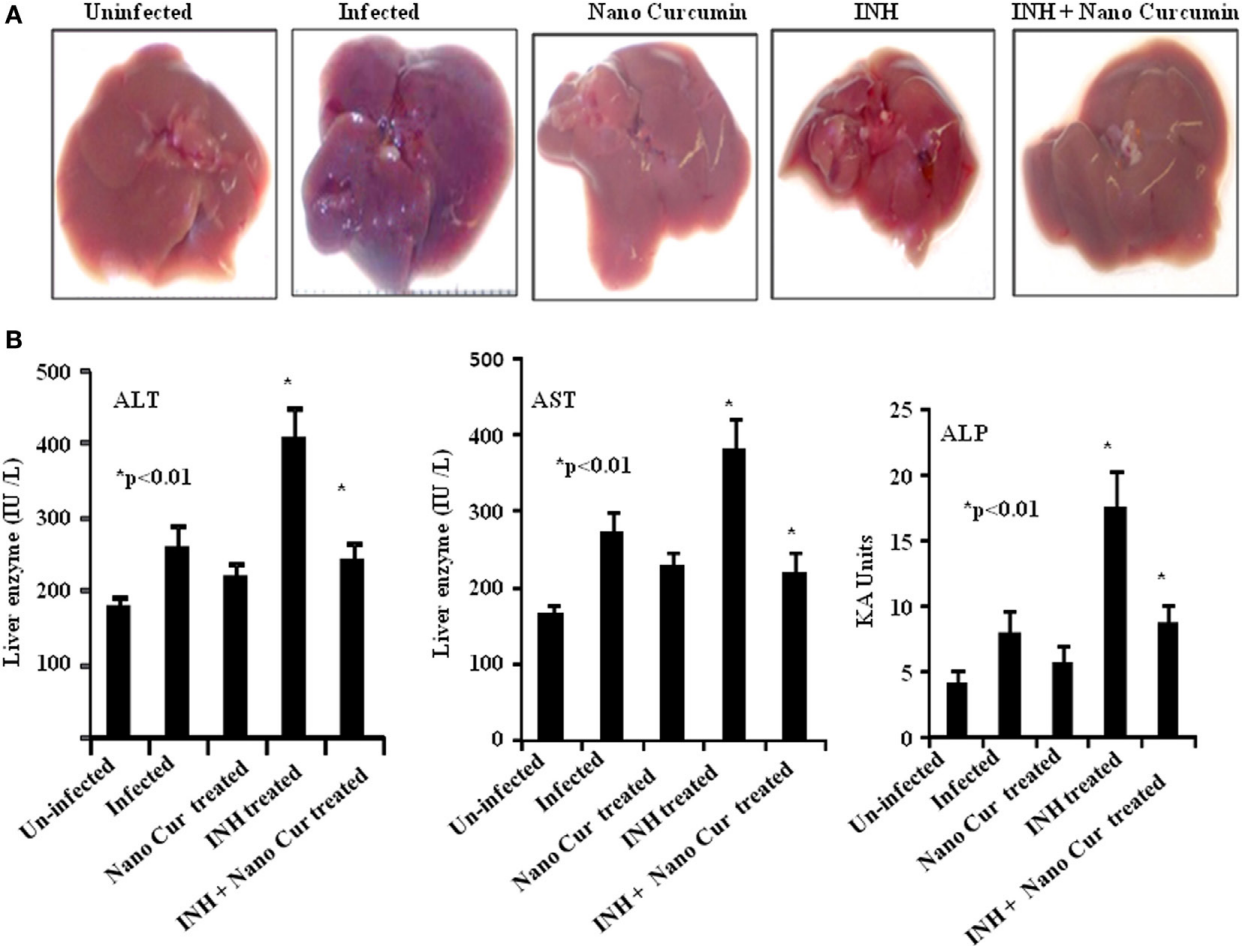
Co-treatment of curcumin nanoparticles with INH reduces inflammatory lesions in liver and hepatotoxicity caused by ATT. **(A)** Macroscopic pathology of liver at different time points after infection. Mice infected with a low-dose aerosol inoculum of *Mycobacterium tuberculosis* H37Rv (~110 colony forming unit/mice) were sacrificed 40 days postinfection, and liver was obtained and photographed to study the comparative changes in gross pathology of the liver. **(B)** Hepatotoxicity. For comparative evaluation of hepatotoxicity in the different experimental groups, we assessed serum alanine aminotransferase (ALT), aspartate aminotransferase (AST), and alkaline phosphatase (ALP) levels in serum at different time points. The figure shows serum ALT, AST, and ALP levels as indicators of hepatotoxicity in different experimental groups at 50 days postinfection.

It is well known that antitubercular antibiotics cause severe hepatotoxicity which results in premature therapy withdrawal by a large number of patients. Curcumin is an antioxidant and exhibits hepatoprotective properties in a variety of conditions (38). Therefore, we examined whether curcumin nanoparticles can overcome the toxicity induced by INH in our study. Our findings indicated that treatment with INH causes profound toxicity as deduced by macroscopic analysis of liver (Figure 4A) and by liver enzyme levels (Figure 4B). As expected, curcumin nanoparticles dramatically reduced inflammatory lesions induced by infection as well as toxicity induced by INH. Taken together, adjunct therapy of curcumin nanoparticles is beneficial not only in promoting sterile immunity and inhibiting disease reactivation but also in preventing hepatotoxicity.

### Curcumin Nanoparticles Enhances Antigen-Specific T Cell Immunity

**Figure 5:**
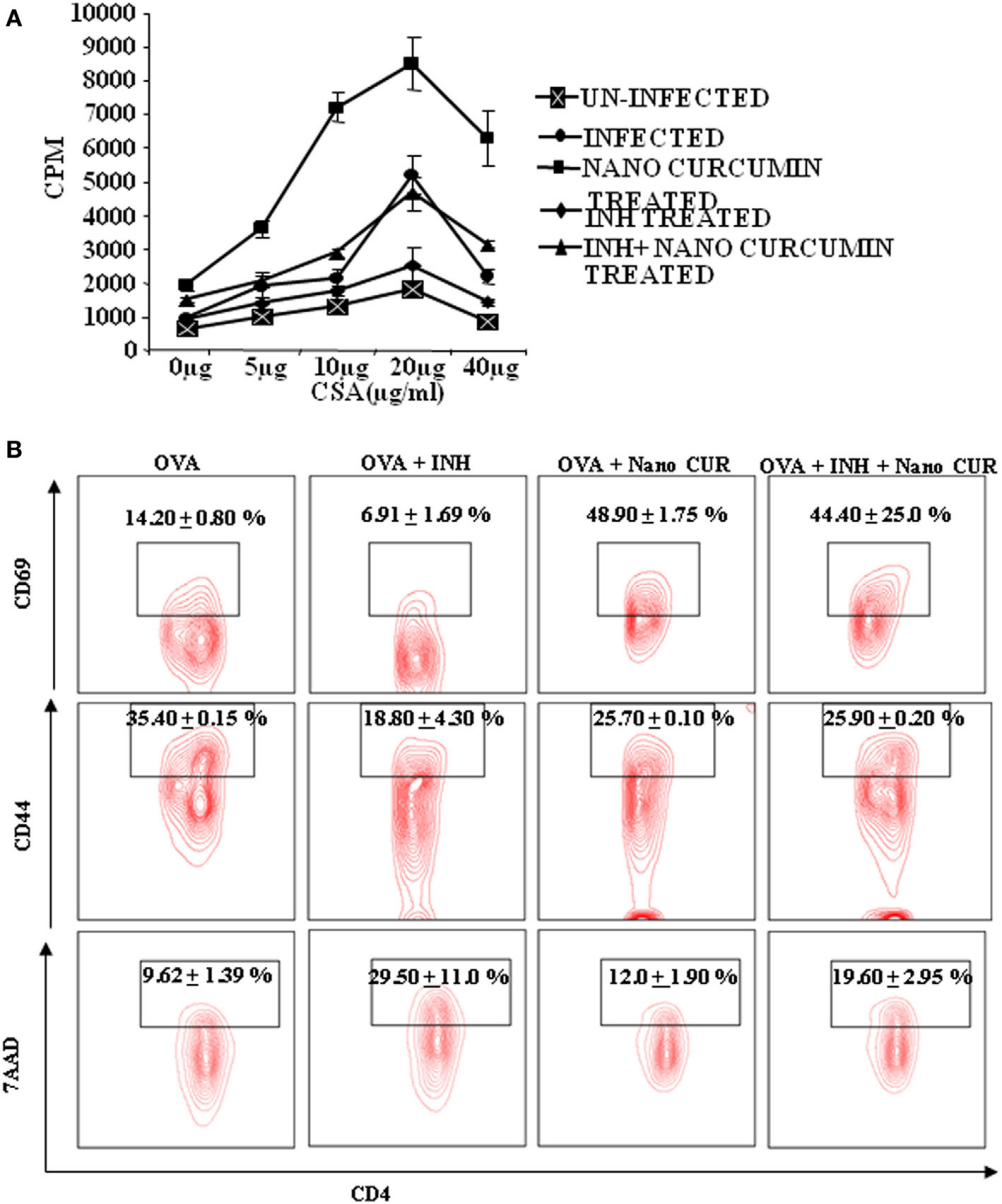
T cell AICD and immune suppression induced by isoniazid can be overcome by curcumin nanoparticles. **(A)** Antigen-specific T cell proliferation. T lymphocytes were isolated from spleens of mice 40 days postinfection, and T cell proliferation assays were performed using tritiated thymidine. *In vitro* T cell proliferation of splenocytes from mice at different time points after infection was compared, after stimulation with *Mycobacterium tuberculosis* H37Rv complete soluble antigen (CSA). Data shown here are representative of five independent experiments with three mice in each group and represent the mean ± SD values. **(B)** Effect of isoniazid and curcumin nanoparticles on the activation of OT-II TCR transgenic T cells in the presence of ovalbumin (OVA) peptide. Splenocytes isolated from TCR Tg mice were cultured in the presence of 1 µg/mL OVA peptide along with 1 µg/mL INH and 10 µg/mL curcumin nanoparticles for 72 h. The cells were then stained and analyzed for T cell activation (CD44 and CD69) and cell death (7AAD) by FACS. The percentage of cells expressing CD44, CD69, or 7AAD among CD4^+^ T cells is shown with mean ± SD. Data shown here are representative of two independent experiments with two replicates in each group.

Next, we sought to examine the mechanisms by which curcumin nanoparticles exhibit their beneficial activities during TB treatment. Previously, we have shown that prolonged use of INH induces apoptosis in antigen-responding T helper (Th) cells and, thus animals treated with INH exhibit increased susceptibility to *M.tb* reinfection and reactivation as compared with untreated animals (22). Similar observations have been reported in human studies as well (23). Therefore, we tested if co-treatment of curcumin nanoparticles with INH has any effects on the nature of the immune response *in vitro*. We stimulated spleen cells isolated from *M.tb* infected animals with *M.tb* derived complete soluble antigen in the absence or presence of INH. Surprisingly, we found that in the presence of INH, antigen-specific proliferation of spleen cells was significantly suppressed (Figure 5A) and curcumin nanoparticles not only restored proliferation but also were capable of alleviating the INH-induced immune suppression. To further investigate whether INH generally induces apoptosis in activated T cells, we activated OVA-specific, OT-II TCR transgenic T cells with OVA peptide in the presence or absence of INH and curcumin nanoparticles. We observed that apoptosis induced by INH in activated T cells was circumvented by curcumin nanoparticles (Figure 5B). These observations indicated that curcumin nanoparticles prevent INH-induced immune dysfunction of T cells *in vitro*. Furthermore, we found that co-treatment with curcumin nanoparticles significantly reduced apoptosis in T cells (Figure 5B) and restored antigen-specific T cell proliferation.

### Curcumin Nanoparticles Induce Enhanced T Cell Responses *In Vivo*

**Figure 6:**
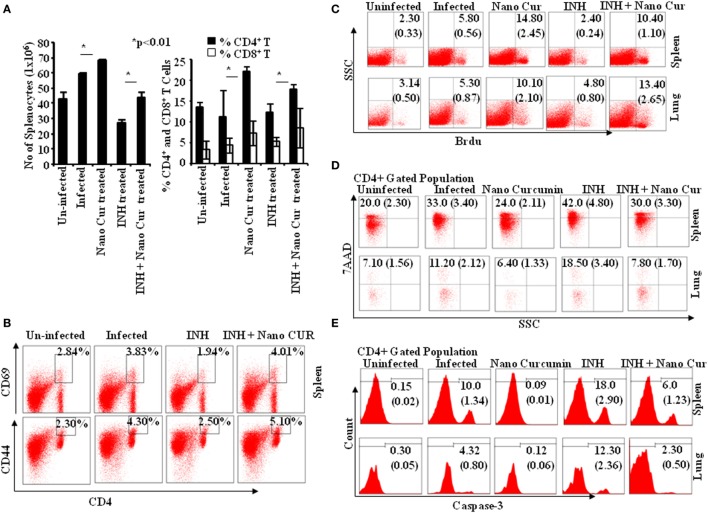
Isoniazid treatment promotes AICD in CD4 and CD8 T cells *via* caspase-3-mediated apoptosis, which can be controlled by combined therapy with curcumin nanoparticles. **(A)** Left panel: total number of splenocytes 40 days postinfection. Total numbers of splenocytes were counted after preparation of single cell suspensions using a hemocytometer. Data shown here are representative of five independent experiments with three mice in each group and represent the mean ± SD values. Right panel: percentage of CD4^+^ and CD8^+^ cells in spleen. Splenocytes were stained with anti-CD4 and anti-CD8 antibodies, and data were acquired by flow cytometry. The percentage of cells expressing CD4 and CD8 is shown in the bar diagram with mean ± SD. Data shown here are representative of three independent experiments with three mice in each group. **(B)** Effect of isoniazid and curcumin nanoparticles on the activation status of T cells *in vivo*. Splenocytes isolated from mice infected and then treated with INH or INH + CURCUMIN nanoparticles for 45 days were surface stained with anti-CD4, -CD44, and -CD69 antibodies, and samples were acquired by flow cytometry. CD4^+^ T cells were gated for CD69 expression, and the percentage of cells expressing CD69 and CD44 among CD4^+^ T cells is shown in the FACS plots with mean ± SD. Data shown here are representative of three independent experiments with three mice in each group. **(C)** *In vivo* T cell proliferation in spleen and lung. To study the status of host T cell proliferation *in vivo* during infection and treatment, 0.6 mg of BrdU in 100 µL PBS was administered intraperitoneally to each mouse for 3 days prior to sacrifice. Cells were then isolated from both lung tissue and spleen of mice and stained with anti-BrdU antibodies. Data were acquired by flow cytometry. The percentage of cells showing BrdU incorporation in different groups at 40 days postinfection is shown in the dot-plot diagram with mean ± SD. Data shown here are representative of three independent experiments with three mice in each group. **(D)** CD4^+^ T cell death. Lymphocytes obtained from spleen and lung tissue of mice sacrificed 40 days postinfection were surface stained with anti-CD4 antibodies followed by 7AAD staining for 20 min prior to acquisition. Samples were acquired by flow cytometry to assess cell death in splenocytes and alveolar lymphocytes. The percentage of cells expressing 7AAD among CD4^+^ T cells is shown with mean ± SD. Data shown here are representative of three independent experiments with three mice in each group. **(E)** Caspase-3 activation. Caspase-3 activation among CD4^+^ lymphocytes of lung and spleen of mice sacrificed 40 days postinfection was estimated by flow cytometry. Data shown here are representative of three independent experiments with three mice in each group.

To obtain insight into T cell responses *in vivo*, we further monitored T cell responses in the murine model of TB. We tested Th cell frequencies in curcumin nanoparticle- and INH-treated animals and found that curcumin nanoparticles not only elevated the total number of splenocytes but also enhanced the frequency and activation of both CD4^+^ and CD8^+^ T cells (Figures 6A,B) during primary infection.

### Curcumin Nanoparticles Rescue T Cells from INH-Induced AICD

To further understand the enhanced T cell response in curcumin nanoparticle-treated animals, we performed BrdU incorporation experiments. We found that proliferation of T cells from lung and spleen of INH-treated animals was profoundly suppressed (Figure 6C), which was restored in animals that received combined therapy with curcumin nanoparticle-treated mice (Figure 6C). Furthermore, our prior *in vitro* observation that curcumin nanoparticles rescue T cells from cell death was also corroborated in the *in vivo* model (Figure 6D). This was most likely due to the capacity of curcumin to inhibit the induction of apoptosis in activated T cells. To further investigate this hypothesis, we studied the status of activated caspase-3 in the T cell population. We found that curcumin nanoparticles inhibit the activation of caspase-3 (Figure 6E), which is critical for the initiation of apoptosis.

## Conclusion

Irrespective of socioeconomic status, nearly all countries are now under threat from drug-resistant (MDR and XDR) TB (1). The discovery of new generations of antibiotics has lagged far behind in the wake of the emergence of drug resistance, and a totally drug-resistant form of TB has already appeared (51–54). Therefore, new ways to thwart the emergence of drug-resistant *M.tb* strains are urgently needed. *M.tb* acquires drug resistance predominantly due to premature treatment withdrawal by patients. The kinetics of *M.tb* clearance with DOTS treatment is bi-phasic. In the initial phase (~2 months) of treatment, the majority of the bacteria are cleared but small numbers (in the latent form) remain, clearance of which requires an additional 4–6 months of treatment (1, 5, 25). During the long phase of treatment, while patients are asymptomatic, patients often withdraw from therapy as they don’t feel the need to continue treatment. A second reason for treatment cessation is prolonged hepatotoxicity (19–21). Finally, long-term treatment does not always lead to complete clearance of the organisms (1).

To avoid the generation of drug-resistant strains new treatments should aim at: (i) shortening the treatment course; (ii) attaining sterile immunity; and (iii) reducing hepatotoxicity. Curcumin is an excellent immunomodulator that promotes antimycobacterial immunity, thereby reducing the length of treatment when combined with conventional antibiotics. This reduction in length of treatment may reduce the possibility for generating drug-resistant variants of TB. Furthermore, elevated immunity helps in attaining complete clearance much faster, which is further beneficial in reducing the possibility of disease reactivation.

Curcumin is an anti-inflammatory agent that exhibits efficacy against several inflammatory and infectious diseases. However, bioavailability of curcumin and metabolic instability under physiologic conditions has hindered its use as a drug. Therefore nanotechnology-based novel drug delivery methods show promise to overcome these challenges. Polymeric nanoparticle-based encapsulation of curcumin (55) or nanoliposome-encapsulated forms of curcumin (56) have been successfully generated. Although such formulations of curcumin have been successfully delivered into cells *in vitro*, loading curcumin onto nanoparticles or trapping curcumin inside of liposomes followed by its controlled release at the desired tissue site has been challenging. In addition, toxicity arising from the long-term use of such formulations of curcumin *in vivo* has not yet been reported. We have therefore used pure curcumin to prepare our nanoparticles, which is chemically identical to natural curcumin as both of them had similar HPLC and NMR profile. However, it is about five times more bioavailable (*C*_MAX_) and circulates for a longer period of time in the blood (*T*_1/2_). Curcumin, being non-toxic, can be given up to 8 g/day to humans (47, 57). Therefore this nanoparticle formulation of curcumin could be used in human subjects at very high doses. Our curcumin nanoparticles are stable and can be administered both orally as well as intraperitoneally and, therefore, have greater potential for therapeutic use under different conditions.

Tuberculosis is a disease with two components, such as microbial infection and inflammation. Therefore, addition of steroids to the conventional antibiotic treatment regimen displays improved efficacy (58). Steroids have several limitations and, therefore, curcumin may serve as both an immunomodulator and an anti-inflammatory agent, which might aid in reducing the length of treatment. Previously, we have demonstrated that antitubercular antibiotics eliminate antigen-responding T cells during treatment leaving animals hyper-susceptible to reinfection (22). This is consistent with the finding that patients treated with antibiotics are hypersusceptible to disease reactivation and reinfection (23, 24). Curcumin prevented apoptosis in antigen-responding T cells induced by antibiotics and would thus be expected to promote improved memory responses upon completion of combined therapy. Interestingly, curcumin is an inhibitor of Kv1.3 (35), which is predominantly expressed by effector memory T (T_EM_) cells (59, 60). Recently, we have shown that inhibition of Kv1.3 by clofazimine dramatically enhances the generation of central memory T (T_CM_) cells, a subset of memory T cells that dictate prolonged efficacy of a vaccine by inhibiting Tem cells (61). Similarly, inhibition of Kv1.3 by curcumin may contribute toward host protection against TB reinfection by expanding the T_CM_ population.

Current antibiotics used in DOTS therapy are toxic, which is a major clinical cause of treatment cessation that can contribute to the generation of drug-resistant TB (15, 51, 52, 62, 63). Curcumin has been shown to alleviate hepatotoxicity in various situations (38). In our study, we also found that combined treatment of nanoparticle-formulated curcumin drastically inhibited hepatotoxicity induced by antibiotics. Therefore, addition of curcumin along with conventional antibiotics provides additional benefit in reducing hepatotoxicity.

Addition of an immunomodulator to conventional antibiotic therapy has beneficial effects in the treatment of TB (64) and has been recommended for effective treatment. Curcumin is an excellent immunomodulator that reduces the length of treatment and helps in attaining sterile immunity when combined with conventional antibiotics. Furthermore, it prevents apoptosis of antigen-responding T cells and heightens memory T cell responses during treatment. As curcumin is an inhibitor of Kv1.3, it alters the ratio of T_CM_:T_EM_, which provides protection against posttreatment infection. In addition, curcumin inhibits hepatotoxicity induced by antibiotics. These beneficial features of curcumin are directed to the host, and its implications are therefore not limited to *M.tb* infection. Taken together, our findings raise enthusiasm for initiating clinical trials to test the safety and efficacy of curcumin nanoparticles as an adjuvant for treatment of TB and other diseases.

### Summary

We generated curcumin nanoparticles, which have a uniform size of ~200 nm and showed extended bioavailability *in vivo*. These curcumin nanoparticles were not only antimycobacterial but also enhanced *M.tb* clearance by promoting antitubercular immunity and thereby reduced the duration of therapy required to achieve sterility. Curcumin nanoparticles induced heightened T cell immunity, which prevented disease reactivation and reinfection. Adjunct therapy of curcumin nanoparticles not only promoted sterile immunity and prevented disease reactivation but also resulted in preventing hepatotoxicity, thereby making it a very suitable adjunct for TB therapy.

## Ethics Statement

All animal experiments were conducted in accordance with guidelines approved by the Institutional Animals Ethics Committee (approval ID: ICGEB/AH/2011/2/IMM-30) of ICGEB, New Delhi, India and Department of Biotechnology (DBT) guidelines, Government of India. At the relevant times after infection with *M.tb*, all mice were humanely sacrificed by asphyxiation in carbon dioxide according to institutional and DBT regulations.

## Author Contributions

SK and GD conceived the project and supervised the research; ST, DS, and GD designed the experiments; DS, ST, SM, SA, and RA performed the experiments; ST, DS, RN, AR, MB, LK, SK, and GD prepared figures and Supplementary Material section; ST, DS, LK, SK, and GD wrote the manuscript. All the authors contributed to final data analysis, discussions, and manuscript preparation.

## Conflict of Interest Statement

The authors declare that the research was conducted in the absence of any commercial or financial relationships that could be construed as a potential conflict of interest.

## Supplementary Material

Figure S1 INH suppresses cytokine production by splenocytes. Splenocytes were cultured and activated with 50 ng/ml phorbol 12-myristate 13-acetate and 750 ng/ml ionomycin overnight, and 10 µg/ml Brefeldin A was added during the last 6 h of culture. Cells were then intracellularly stained with anti-IL4, -IFN-γ, -IL17, -IL9, -IL10, -IL12, -TNF-α, -IL6, or -IL22 antibodies and appropriately labeled control antibodies. Cells were acquired by a flow cytometer. Data are shown as mean ± SD, and Student’s *t*-test was done for estimating significance between two groups.

Figure S2 Cytokine production by splenocytes from treated mice that were reinfected with *Mycobacterium tuberculosis*. Splenocytes were cultured and activated with 50 ng/ml phorbol 12-myristate 13-acetate and 750 ng/ml ionomycin overnight, and 10 µg/ml Brefeldin A was added during the last 6 h of culture. Cells were then intracellularly stained with anti-IL4, -IFN-γ, -IL17, -IL9, -IL10, -IL12, -TNF-α, -IL6, and -IL22 antibodies and appropriately labeled control antibodies. Cells were acquired by a flow cytometer. Data are shown as mean ± SD, and Student’s *t*-test was done for estimating significance between two groups.
