# Supplementary figures and images for: Nanoparticle-Formulated Curcumin Prevents Posttherapeutic Disease Reactivation and Reinfection with Mycobacterium tuberculosis following Isoniazid Therapy
Source: Front Immunol. 2017 Jun 30;8:739. doi: 10.3389/fimmu.2017.00739 (PMC5491555; doi:10.3389/fimmu.2017.00739)

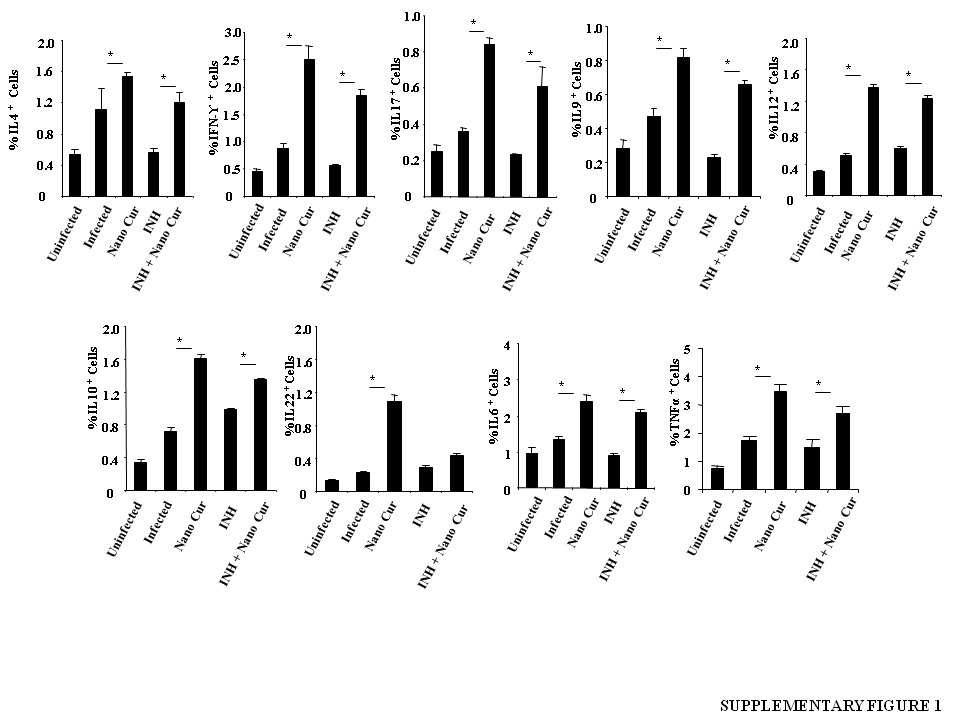

Supplement: Figure S1 — INH suppresses cytokine production by splenocytes. Splenocytes were cultured and activated with 50 ng/ml phorbol 12-myristate 13-acetate and 750 ng/ml ionomycin overnight, and 10 µg/ml Brefeldin A was added during the last 6 h of culture. Cells were then intracellularly stained with anti-IL4, -IFN-γ, -IL17, -IL9, -IL10, -IL12, -TNF-α, -IL6, or -IL22 antibodies and appropriately labeled control antibodies. Cells were acquired by a flow cytometer. Data are shown as mean ± SD, and Student’s t-test was done for estimating significance between two groups. [file Image_1.TIF]

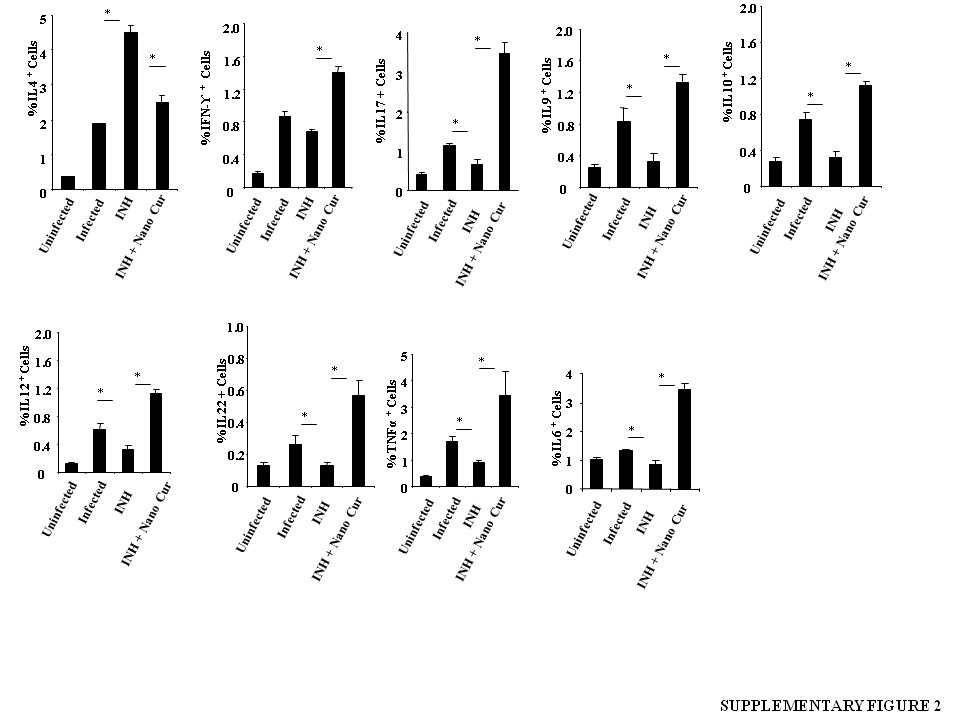

Supplement: Figure S2 — Cytokine production by splenocytes from treated mice that were reinfected with Mycobacterium tuberculosis. Splenocytes were cultured and activated with 50 ng/ml phorbol 12-myristate 13-acetate and 750 ng/ml ionomycin overnight, and 10 µg/ml Brefeldin A was added during the last 6 h of culture. Cells were then intracellularly stained with anti-IL4, -IFN-γ, -IL17, -IL9, -IL10, -IL12, -TNF-α, -IL6, and -IL22 antibodies and appropriately labeled control antibodies. Cells were acquired by a flow cytometer. Data are shown as mean ± SD, and Student’s t-test was done for estimating significance between two groups. [file Image_2.TIF]
